# Gibbon strategies in a food competition task

**DOI:** 10.1038/s41598-021-88804-5

**Published:** 2021-04-29

**Authors:** Alejandro Sánchez-Amaro, Robert Ball, Federico Rossano

**Affiliations:** 1 Department of Cognitive Science, University of California San Diego, La Jolla, USA; 2 Department of Comparative Cultural Psychology, Max Planck Institute for Evolutionary Anthropology, Leipzig, Germany; 3 The Graduate Center, City University of New York, New York, USA

**Keywords:** Social evolution, Animal behaviour

## Abstract

Social primates face conflicts of interest with other partners when their individual and collective interests collide. Despite living in small, primarily bonded, groups compared to other social primates, gibbons are not exempt from these conflicts in their everyday lives. In the current task, we asked whether dyads of gibbons would solve a conflict of interest over food rewards. We presented dyads of gibbons with a situation in which they could decide whether to take an active role and pull a handle to release food rewards at a distance or take a passive role and avoid action. In this situation, the passive partner could take an advantageous position to obtain the rewards over the active partner. Gibbons participated in three conditions: a control condition with no food rewards, a test condition with indirect food rewards and a test condition with direct food rewards. In both test conditions, five rewards were released at a distance from the handle. In addition, the active individual could obtain one extra food reward from the handle in the direct food condition. We found that gibbons acted more often in the two conditions involving food rewards, and waited longer in the indirect compared to the direct food condition, thus suggesting that they understood the task contingencies. Surprisingly, we found that in a majority of dyads, individuals in the active role obtained most of the payoff compared to individuals in the passive role in both food conditions. Furthermore, in some occasions individuals in the active role did not approach the location where the food was released. These results suggest that while gibbons may strategize to maximize benefits in a competitive food task, they often allowed their partners to obtain better rewards. Our results highlight the importance of social tolerance and motivation as drivers promoting cooperation in these species.

## Introduction

Social animals constantly face conflicts of interest with other group members. In primates, decisions about different travel routes, group defense, or access to limited resources may result in divergent preferences for individuals and hence potential conflicts between group members^1–6^. To resolve these conflicts, primates may engage in different sets of strategies including dominance, mutual cooperation or majority rules depending on factors such as group size and social organization^7^. For instance, Lar gibbon (*Hylobates lar*) females lead travel routes and access high value rewards before males despite the lack of a clear dominance of one sex over the other^8^. In olive baboons (*Papio anubis*) instead, travel routes seem to be driven by majority rule despite their highly hierarchical social system^5^. However, while these observations allow us to understand different aspects of primate behavior, it is often difficult to dissect the factors contributing to specific behavioral patterns in nature. Accordingly, experimentally controlled studies with primates can shed crucial light on the decision-making strategies underlying the observed behavior.

To that end, over the last years numerous studies have adapted game theory models to explore the strategies that different primates use to overcome social dilemmas in which their interests come into conflict^9–12^. For instance, computerized tasks have been used to present primate species including chimpanzees, capuchin monkeys and rhesus macaques with economic games borrowed from the game theory literature^13–15^. These studies have found that, in general, primates can converge to a Nash Equilibrium (i.e. the optimal outcome from an interaction given the strategy of your partner) during coordination and conflict games between dyads. Using a different approach, other researchers have presented great apes, mostly chimpanzees (*Pan troglodytes*), with non-computerized social dilemmas in which apes had to decide between different physical actions to obtain rewards from an apparatus. In general, these studies have found that chimpanzees and bonobos develop strategies with the aim to outcompete their partners and obtain the most from the social dilemma, either through monopolization of rewards^11^, by waiting for their partner to act before them^9,10,16^ or by influencing them to change their strategy^12^.

The use of game theory models to explore how different primate species coordinate actions for mutual goals, as well as how they overcome conflicts of interest, is a growing field in comparative psychology^15^. For example, a very recent study presented squirrel monkeys, a primate species that rarely cooperates in nature, with a set of computerized social dilemmas previously presented to more cooperative species such as the capuchin monkeys (*Cebus apella*). Vale and colleagues^17^ found that squirrel monkeys (*Saimiri boliviensis*) behaved similar to capuchin monkeys in cooperative scenarios such as the assurance game. Squirrel monkeys were able to coordinate with their partner to obtain the highest rewards. In another recent study, Sánchez-Amaro and colleagues^18^ presented for the first time pairs of common marmosets (*Callithrix jacchus*) with a social dilemma modeled after the snowdrift game^10,19^. In this study, marmosets could access an unequal reward distribution in the form of a rotating tray. In the social dilemma condition, the preferred reward could only be obtained by waiting for the partner to act, with the risk that if none of the two accessed the tray they would both lose the rewards. The authors explored whether cooperative breeding marmosets would engage in more cooperative strategies than great apes due to their natural tendency to act prosocially toward others in different contexts including food sharing. They found similarities between the strategies of marmosets, chimpanzees and bonobos to maximize benefits (e.g. waiting for the partner to act before them). They also found sex differences between the strategies of females and males, where the former were less willing to act—a strategy that allowed females to maximize rewards over males. The marmoset results fit the natural history of this species in which males usually donate food to females in food sharing tasks^20^, presumably to overcome the high energetic costs of female reproduction^21,22^. However, we still know very little about the socio-cognitive strategies that other primate species may develop to deal with similar conflicts of interest.

Perhaps surprisingly, one of the primate families we know less about in terms of their socio-cognitive abilities are the gibbons (family *Hylobatidae*). These small apes are key species in the sense that they are closely related to both cercopithecoids and great apes^23^. Furthermore, unlike any other ape species, gibbons primarily live in small groups mainly composed of a bonded male–female pair and their dependent kin^24^. Thus, the study of their socio-cognitive abilities is very relevant to understand whether some cognitive traits observed in other primates are shared across anthropoids or, instead, evolved independently in diverse lineages. Up to date, the study of gibbons has mainly focused on elucidating aspects of their biology, ecology and phylogeny^25,26^, with little work assessing their socio-cognitive abilities in experimentally controlled settings. For instance, in a recent literature review on primate cognitive studies published by the Manyprimates initiative^27^, it was found that gibbons only appeared in 2 of the 574 studies surveyed between 2014 and 2019.

Gibbons may have been excluded from cognitive studies due to difficulties securing a sufficient sample size to conduct experimental studies or due to their limited motivation to participate in cognitive tasks^28–30^. While limited sample size is a problem that a majority of comparative psychologists and primatologists face when studying primate behavior, a lack of motivation is often the product of experimental designs and methods not suited to the biology of the species. In the case of gibbons, early work by Beck^31^ already showed, for example, that adaptations to the way gibbons could interact with an apparatus (e.g. lifting the access to the ropes instead of leaving them on the ground on a flat surface) improved their participation and performance when compared to previous studies^32,33^.

Furthermore, as Liebal emphasizes^30^, it has been sometimes assumed that gibbons are less interesting than other primate species due to their relatively simpler social system based on pair-living, which assumes low socio-cognitive abilities in relation to other primates. However, mounting evidence over the last 20 years has challenged the assumption that gibbons are truly monogamous^24,25,34–36^. Some gibbon species have been found to engage in extra-pair copulations^37^ and a number of studies have reported different group structures in addition to pair-living^36,38–41^. In other words, the social organization of gibbons is more flexible than originally described. Furthermore, the fact that gibbons live in reduced groups does not necessarily presuppose a lack of social complexity. According to Freeberg^42^, pair-bonded individuals form more complex and intense relationships than those living in large polygamous groups—possibly because they are more interdependent. In other words, social complexity is not only a matter of group size but of relationship quality. It is thus possible that gibbons would engage in more prosocial strategies between closely bonded partners than other primates living in larger groups when conflicts of interest take place.

Nevertheless, despite the lack of studies on gibbon socio-cognitive abilities in relation to other primate species^27^, researchers have made significant advancements on this area. For instance, it has been investigated whether gibbons recognize themselves in the mirror although the findings remain inconclusive (see^30^ for review). Furthermore, there has been extensive research on the abilities of gibbons to follow the gaze of others to discover an unseen object. Researchers have found that gibbons are able to shift their gaze in response to an experimenter gaze shift but it remains unclear whether gibbons are taking the perspective of the experimenter into account, including her mental states^43–47^.

To evaluate whether gibbons take into account the perspective of others, a recent study presented gibbons with a competitive scenario in which they could only retrieve uncontested rewards when the experimenter did not orient his body, head or eyes towards the rewards. The experimenters found that gibbons avoided the contested table by paying attention to the orientation of the body and the head of the experimenter but not to his eyes^48^. This later result suggests that, in line with previous socio-cognitive studies in other primate species^49–51^, gibbons may perform better in competitive settings compared to neutral ones.

However, in previous gaze-following studies with gibbons the participants in the interaction were the human experimenter and the ape. Gibbons did not interact with conspecifics. Considering the competitive task presented by Sánchez-Amaro and colleagues^48^ as an example, the experimenter and the gibbon faced a conflict of interest every time the gibbon approached the contested table since the experimenter and the gibbon competed for the same food reward. Therefore, one open question is how dyads of gibbons would solve conflicts of interest in a more naturalistic context. That is, when they need to deal with other conspecifics over access to resources such as food rewards.

To answer this question, we presented dyads of gibbons with a simplified version of a social dilemma resembling a snowdrift game^19^. In this game two individuals can decide whether to cooperate or defect to obtain a benefit. If they engage in mutual cooperation they share the cooperation costs and both benefit. Alternatively, an individual can defect and obtain the highest benefit if the partner cooperates. However, if they both decide to defect they both lose. Thus, in the snowdrift game unilateral cooperation is still preferable over mutual defection—it is better to pay the cooperation costs even if your partner defects. Importantly, our setting maintains all core aspects of a snowdrift game with the exception that individuals cannot engage in mutual cooperation (see^10^ for a similar design), although they could cooperate equally by taking turns across trials. In that sense, our game could also be understood as a 2-person Volunteer’s Dilemma^11,52^.

This is the first time a game theory model has been implemented to shed light on the nature of gibbon socio-cognitive abilities. To recreate the social dilemma, we presented dyads of gibbons with the possibility to individually pull from a handle (a PVC pipe). By pulling the handle, a rope would lift a release mechanism and rewards would fall at a distance from the handle. The dilemma would occur if the partner that did not pull took advantage of the situation and position herself in front of the release mechanism—with the possibility to benefit more than the individual who pulled the handle. Importantly, individual roles were not assigned by the experimenter. Gibbons could decide whether to cooperate or not by pulling the handle. For the sake of clarity, we will characterize the individual who took an active role on a given trial and pulled as the actor, and the individual who took the passive role on a given trial and waited as the passive partner.

Dyads of gibbons were presented with three conditions varying in the number of available rewards: a test condition with direct food rewards in which the actor could obtain one extra reward attached to the handle while releasing five rewards at a distance from herself; a test condition with indirect food rewards in which the actor did not obtain an extra reward from pulling the handle but could still release the five rewards at a distance and a no food control condition with no rewards involved. Importantly, both the actor and the passive partner could benefit from the five rewards. The extra reward inside the handle was mostly accessible to the actor but the passive partner could theoretically obtain it as well. For the sake of clarity, we will refer to the three conditions succinctly as direct food test condition, indirect food test condition and no food control condition.

We expected gibbons to act more often in the direct and the indirect food test conditions compared to the no food control condition. This would show that gibbons understood the contingencies of the game. Furthermore, given that gibbons were living with close partners one possibility is that they would share the potential costs of cooperation. By cooperative costs we refer to the possibility of losing rewards due to the distance they had to cover and the time lost to return to the location where the food was released, especially if their passive partners took an advantageous position to obtain the released rewards.

If, in contrast, gibbons would react competitively as other great ape species did in previous social dilemmas, we would expect them to try to maximize their food rewards by hesitating to act and by taking advantage of their passive role—placing themselves in front of the release mechanism. In that sense, we would expect passive partners to benefit from their position and obtain more rewards than the actors. Furthermore, we would also expect gibbons to be more likely to act in direct food test trials given that they could easily obtain at least one direct reward from their actions.

## Results

### Rate of participation and latencies

**Figure 1:**
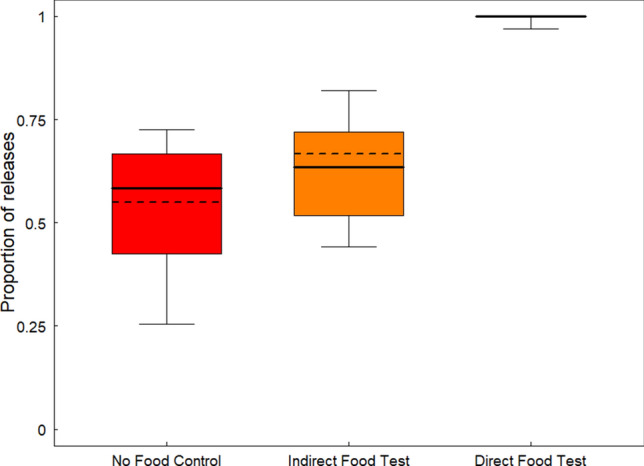
Proportion of trials in which food was released. The box plot represents the median and q1 and q3 quartiles. The dotted line represented the fitted values of the model.

Gibbons decisions to pull the handle and release the food container were affected by the condition presented (GLMM: *χ*^2^ = 121.48, df = 2, *p* < 0.001, N = 532, see the Electronic Supplementary Materials (ESM) Model 1). Pair-wise comparisons indicated that gibbons pulled significantly more often in the direct food test condition compared to the indirect food test condition (GLMM: *χ*^2^ = 121.48, df = 2, *p* < 0.001, N = 532, CI [4.03, 14.81]) and the no food control condition (GLMM: *χ*^2^ = 121.48, df = 2, *p* < 0.001, N = 532, CI [4.7, 15.49]). Gibbons pulled in all direct food test trials but one while in indirect food test trials and in no food control trials gibbons pulled less frequently (62.5% and 53.1% respectively). Interestingly though, gibbons pulled significantly more often in the indirect food test condition compared to the no food control condition (GLMM: *χ*^2^ = 121.48, df = 2, *p* = 0.048, N = 532, CI [-0.99, 0.002]), suggesting that the potential release of food rewards at a distance also motivated gibbons’ decisions and that they could distinguish between the three conditions presented (Fig. 1).

Next, we found that gibbons’ latencies to pull were also affected by the condition presented (see ESM Model 2 Fig. S1). That is, in those trials in which individuals pulled (71.8% of trials), they clearly acted faster in the direct food test condition (when one piece of food was directly accessible from the handle, average latency 5.6 s) compared to the indirect food test condition (coxme, HR = 4.98, *p* < 0.001, N = 532, CI [3.74, 6.29], average latency 17.4 s) and to the no food condition (coxme, HR = 6.39, *p* < 0.001, N = 532, CI [4.66, 8.05], average latency 17.9 s). Despite gibbons being more likely to pull in the indirect food test condition compared to no food control condition, their latencies to act did not statistically differ between those two conditions in those trials in which they accessed the rope (coxme, HR = 0.78, *p* = 0.077, N = 532, CI [0.659, 1.04]). We also found that gibbons tended to act faster across sessions (coxme, HR = 1.09, *p* = 0.003, N = 532, CI [1.01, 1.14]) but increased their latencies to act within sessions (coxme, HR = 0.97, *p* = 0.03, N = 532, CI [0.96, 1]).

### Strategies by actors and passive partners

**Figure 2:**
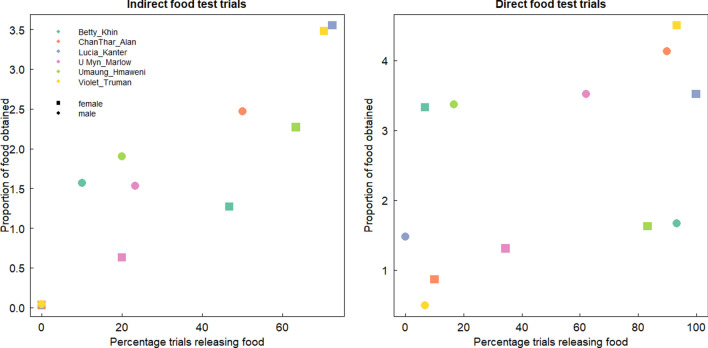
Proportion of food obtained per individual (out of the 5 released rewards) in relation to the percentage of indirect food test trials (left) and direct food test trials (right) in which they released the rewards. Each color represents one dyad. Squares represent females and dots represent males.

We also analyzed whether actors received fewer blueberries (out of the five blueberries released at a distance) than passive partners. To answer this question, we analyzed whether the individual role (actor or passive partner) would predict the amount of released food that individuals obtained across conditions. Surprisingly, we found a significant two-way interaction between condition and role suggesting that passive individuals obtained less food than actors and that this difference was especially salient in indirect compared to direct food test trials (GLMM: *χ*^2^ = 7.57, df = 2, *p* = 0.006, N = 572, CI [-1.64, -0.19], see ESM Model 3 Fig. S2). Therefore, actors were still able to obtain the majority of the five released rewards in both conditions. Furthermore, actors obtained the extra rewards in 87% of the direct food test trials in which they acted. In line with this finding, Fig. 2 shows the percentage of trials in which individuals release food against the number of rewards that each individual obtained in indirect and direct food test trials. Interestingly, in only one dyad (Betty and Khim Maung Win) the more active individual obtained less benefits than the passive partner in both conditions. We also found significant effects of our control variables age and length of dyad (see ESM Model 3) suggesting that younger individuals obtain more food and that dyads who have been longer together distributed the five rewards more equally.

Next, we evaluated whether passive gibbons (those not releasing the food on a given trial) acted strategically by placing themselves in front of the ramp at the moment the actor released the five food rewards and whether they would take advantage of their position in direct food test trials compared to indirect food test trials given that the actor gibbon would spend some time retrieving the food baited inside the handle.

There were 287 instances in which the food was released by an actor in the direct and indirect food test conditions. In 235 (81.9%) of those trials the passive individual was not in front of the ramp by the time the food was released. The passive individual only placed herself in front of the ramp by the time the food was released in 52 trials (18.1%) (32 direct food test trials (61.5%) and 20 indirect food test trials (38.5%)) and there were no differences between conditions (GLMM: *χ*^2^ = 0.31, df = 1, *p* = 0.58, N = 287, see ESM Model 4). Furthermore, in 49 of those 235 trials, the partner was within a meter from the subject by the time the subject released the food (40 of 145 direct food test trials (27.6%) and 9 of 90 indirect food test trials (10%)).

We also explored whether gibbons would take an advantageous position after the food had been released. For that we measured whether the passive individual was in front of the ramp by the time the actor arrived at the location where the food had been released. After releasing the food, the actor moved to the food location in 237 of those 287 trials (82.6%). Passive individuals were in front of the ramp by the time the actor arrived in 76 occasions (32.1%). We found no differences between conditions (GLMM: *χ*^2^ = 0.69, df = 1, *p* = 0.41, N = 237, see ESM Model 5).

We found that actors did not approach the released food in 50 trials in which they released it. A majority of those trials were direct food test trials (37 of 50, binomial test *p* < 0.001). This makes sense since actors spent some time obtaining the food reward placed inside the handle. Nevertheless, in a great majority of these trials (44 of 50 trials; 88%) the actors ended up obtaining the food reward from the handle before the passive individual finished her last reward. Interestingly though, this behavior predominantly occurred in three of the six dyads. Furthermore, we only found one case in which the actor showed an intention to approach the partner by getting closer to him.

### Social measurements: displacement and cofeeding events

We examined whether displacement and cofeeding events differed between direct and indirect food test conditions. We only found 13 displacements out of 287 trials (4.5%). In a majority of displacement events the passive was already in front of the food when the actor arrived (11 of 13) but only in 3 trials the partner was already there from the moment the food was released. Furthermore, in 10 of 13 trials the active individual displaced the passive one and 10 of these 13 displacements occurred in test trials. Displacements were found in five of the six dyads, ranging from 2 to 5 instances per dyad. Not surprisingly, displacements occurred more often (5 times) in the most recently formed dyad.

Finally, we found cofeeding in 44 trials out of 287. A majority of cofeeding events occurred during direct food test conditions (27 of 44; 61%). However, we found that cofeeding did not significantly vary between direct and indirect test conditions (GLMM: *χ*^2^ = 1.58, df = 1, *p* = 0.21, N = 287, see ESM Model 6). Cofeeding was observed in five of the six dyads, ranging from 2 to 22 instances per dyad. In line with the displacements results, cofeeding never occurred in the most recently formed dyad.

## Discussion

The results of the study suggest that dyads of gibbons managed to solve a competitive food task where one dyad member had the opportunity to pull and activate a release mechanism containing five potential food rewards for both partners. When gibbons had the opportunity to obtain an extra reward from their actions (i.e. in our direct food test condition), they almost always acted, avoiding mutual defection. In contrast, in an indirect food test condition where gibbons could not obtain the extra rewards from their pulling efforts, their likelihood to pull and release potential food rewards dropped significantly in comparison to the direct food test condition. In our opinion, two primary reasons could explain this pattern of results.

First, it is possible that gibbons were more motivated to pull when they could directly benefit from the extra reward inside the handle. Similarly, gibbons would have significantly pulled more often in the indirect food test condition compared to the no food control because they could still obtain some rewards. Clearly, gibbons showed that they did not just act for the sake of pulling the rope. Most likely, their actions were motivated by the prospect of obtaining rewards, especially when those were easier to access.

While this reason is very plausible, it does not necessarily account for why gibbons did not pull for 90 s in almost 40% of the indirect food test trials. This is especially surprising if we consider that actors benefitted more than passive partners in this condition. In other words, gibbons did not seem to interpret the situation as beneficial for actors. If that were the case, we would have expected gibbons to pull more often in indirect food test trials. Thus, the second possible explanation for our results is that gibbons interpreted the situation as a conflict of interest and hesitated to pull to avoid losing rewards in favor of the passive partner. This interpretation would be in line with previous findings in chimpanzees, bonobos and common marmosets^10,16,18^. Thus, given the two non-mutually exclusive explanations, it remains unclear whether gibbons defected in indirect food test trials due to a reduced motivation to act because they could not access the extra reward attached to the handle (but still could benefit from the five rewards) or because they wanted to avoid the possibility of losing rewards to their partner.

So far, we have mainly discussed gibbons’ decisions whether to pull or not. The next question concerns whether gibbons strategized when they took a passive role. In other words, did they try to maximize their own rewards when their partners pulled? The main source of potential conflict between participants lied on the possibility that passive individuals could position themselves in front of the release mechanism during direct and indirect food test trials. In direct food test trials, this could be particularly beneficial for passive partners given that actors could lose some valuable seconds trying to obtain the extra food reward attached to the handle. However, we found that actors actually obtained most of the rewards in both conditions, with a special advantage over passive partners during indirect food test trials. In fact, in only one dyad of gibbons the passive individual obtained more rewards than the actor in both test conditions. Furthermore, passive individuals rarely took advantage of the situation (they positioned themselves in front of the release mechanism by the time the actor pulled in 18.1% of all the direct and indirect food test trials) and they did not distinguish between conditions. That is, during direct food test trials passive partners did not benefit from the time that the actors spent trying to obtain the reward located inside the handle. In that sense, gibbons did not interpret the situation as a social dilemma in which they could benefit more than their partner. Yet, gibbons also did not solve this task cooperatively. For that to be the case, dyad members would have needed to benefit more or less equally on both conditions and they would have not hesitated to manipulate the handle in indirect food trials. It is also very unlikely that gibbons’ decisions were driven by proactive prosocial motivations such as releasing food rewards to favor their dyad members given that actors benefited the most from their own actions.

One possible explanation to understand why actors obtained more rewards than passive partners is that both individuals approached the handle during direct food test trials to obtain the reward, although only one individual pulled the handle. This occurred in 27.6% of the direct food test trials in which an individual pulled the handle. This possibility could partially explain why passive gibbons rarely position themselves in front of the release mechanism during direct food test trials, but it cannot explain why in indirect food test trials they did not take advantage of their passive role. In fact, in indirect food test trials passive partners approached the handle in 10% of trials. An alternative explanation is that the dominant individual pulled and obtained the majority of rewards. However, there are numerous reasons to suggest that a dominance component cannot fully explain our pattern of results. First, only three of 12 individuals released rewards in less than 20% of the direct and indirect food test trials. The result suggests that although they did not solve the task cooperatively, in half of the dyads both individuals exchanged roles relatively often. Furthermore, our results are also in line with literature suggesting that there is no clear dominance of one sex over the other^53^ and that gibbons engage in food sharing (i.e. tolerating others to actively take from them) in both captive^54–56^ and natural settings^57,58^. Importantly, conflict avoidance does not seem to be relevant here either. Cofeeding events occurred three times as often than displacements events, suggesting that individuals were usually tolerant with each other, as it has been observed in other populations. Relatedly, gibbons never hoarded the blueberries. They ate them as soon as they got them. Furthermore, these tolerance may be mediated by the length of time that dyads had spent together. In our study, the length of dyad predicted how equally gibbons divided the five food rewards. A third alternative explanation is that once a gibbon decided to manipulate the handle, the other one totally disengaged from the task, as if acknowledging a sort of property claim. This could explain why passive partners did not take an advantageous position in front of the release mechanism and why, as a consequence, actors did not face a social dilemma in many trials. After all, actors benefit more than passive partners despite the costs to pull the rope, understood as the possibility to lose rewards due to the distance they had to cover and the time lost to return to the location where the food was released. Nevertheless, this is unlikely because passive individuals still obtained a significant fraction of the food rewards (38% of rewards across conditions).

All these previous arguments cannot fully explain why passive partners rarely took advantage of their position, especially given how successful this strategy was: passive partners obtained almost 75% of rewards when they positioned themselves in front of the release mechanism by the time the actor manipulated the handle. We thus propose that passive gibbons did not always take advantage of their partner actions because of the combined processes of motivation from the side of the actor and a general high level of social tolerance towards inequities. That is, individuals that were more motivated to obtain food and more attentive were also more likely to take the actors’ role in our task. Through participation, they could become more aware of the situation as a whole and react faster to obtain the rewards despite the potential costs of pulling the rope. This gave them an advantage over their passive partners, whom at the same time tolerated actors obtaining the majority of rewards (as if actors had called “dibs” on the rewards). Importantly, tolerance towards reward inequities also came from the perspective of the actors. Indeed we found that in fifty trials individuals from five of the six dyads (in all dyads excepted in the most recently formed) seemed to adopt a prosocial attitude towards their passive partners by letting them access all the released rewards.

Future studies should improve different aspects of our setup to continue exploring gibbons’ decision-making strategies when individuals’ interests collide. The main weakness of our design is that we were not able to separate dyads of gibbons before the experimental sessions. In that sense we could not train them with the different task contingencies as it is usually the case in this type of settings^10,59,60^ but see^61–63^. It is thus possible that some individuals were more skillful than others, and that might have affected our results. Nevertheless, all individuals approached the apparatus and obtained rewards and only one never pulled from the handle during the course of the study. Moreover, despite the fact that gibbons distinguished between conditions with food (direct and indirect food test trials) and the no food control, their latencies to act did not differ between the indirect food test trials and the no food control trials. This could be interpreted as strategic behavior in the indirect food test trials if the gibbons were waiting for their partners to act. It is possible that with longer trials we would have found a significant difference between gibbons’ latencies in indirect food test trials and no food control trials. In that sense, future changes in the trial time or the food rewards can better assess whether gibbons strategize to solve conflicts of interest. Relatedly, it is possible that gibbons were more motivated to act during direct food test trials compared to indirect food test trials because there were more rewards in play (6 vs 5 rewards). It has been found that at least great apes are able to distinguish between these two amounts when they are presented at the same time^64^. To our knowledge, however, gibbons have never been tested with these quantities. Nevertheless, in our scenario gibbons did not necessarily need to discern between these two quantities because the two amounts were never presented at the same time. Thus, although it is possible that this difference could have played a role in their performance, it is more parsimonious to think that their motivation to pull in direct food test trials was due to the high probability to eat the extra reward while pulling the handle. Future studies may use different reward constellations varying in quantity and/or quality to continue shedding light on gibbon socio-cognitive performance. Finally, given the quasi-experimental nature of our task, we did not always capture the social dilemma scenario we envisioned. Future tasks should implement designs in which cooperative acts are clearly costly for those individuals willing to volunteer. In addition, given our restricted sample size we could not test species differences or the presence of individual biases (e.g. bias leading individuals towards becoming actors).

The present study advances our understanding of how tolerance may allow primates to solve potential conflict over food rewards^65^. In our study gibbons exhibit high degrees of social tolerance (in particular in the form of reactive, rather than proactive prosociality^66^). Passive partners tolerate that actors obtain higher benefits in a majority of trials while actors often actively forego opportunities to maximize rewards (e.g. the actor does not try to obtain any of the released rewards). Relatedly, gibbons engaged in cofeeding events relatively often. One possibility is that such a high degree of social tolerance towards conspecifics results from gibbons’ unique pair-living social system compared to other great apes, although future studies should inspect this relationship in more detail. Overall, the inclusion of gibbons in studies exploring the nature of primate socio-cognitive abilities is critical. It will help to elucidate the nature of our prosocial motivations and their relationship to specific socio-ecological pressures and ultimately to understand how they have evolved since the last common ancestor with all living apes.

## Materials and methods

### Subjects

Seven eastern hoolock gibbons (*Hoolock leuconedys*)**,** two pileated gibbons (*Hylobates pileatus*), two northern white cheeked gibbons *(Nomascus leucogenys*), and one siamang (*Symphalangus syndactylus*) participated in the current study (total N = 12, 6 M: 6F, age in years = 13.98 ± 6.99). See Table S1 in the ESM for info about the subjects.

All subjects were housed at the Gibbon Conservation Center (GCC) in Santa Clarita (CA, USA). They lived in dyads of the same species, with the exception of one eastern hoolock gibbon, who was housed with one siamang. Only two dyads were potentially breeding pairs—the other four dyads included individuals on contraceptives. Subjects were tested together in their enclosure and participation was voluntary. Subjects were fed multiple small meals per day outside of testing food. Testing took place between meals to ensure motivation. Water was accessible ad libitum.

### Setup and materials

One experimenter (hence E1) interacted with the apes during a test session while a second experimenter recorded the session and scored the subjects behavior (hence E2). Each experimenter tested half of the dyads. We used high quality rewards (blueberries) that would be easily visible to the subjects. Blueberries were not part of their daily diet but were sometimes presented as enrichment in puzzle feeders and were highly desirable for all gibbons housed at the GCC.

**Figure 3:**
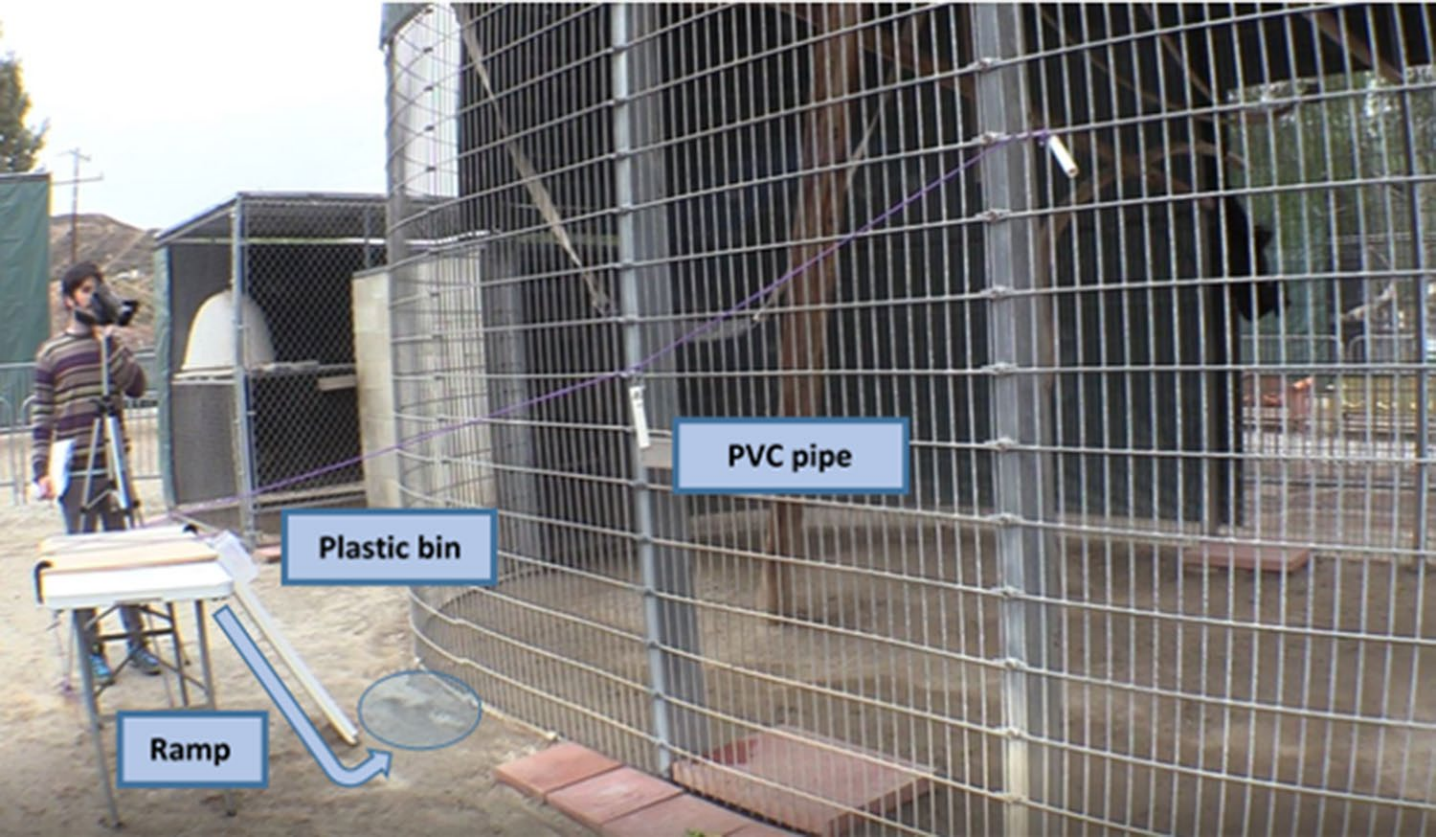
Details of the study setting. The arrow and the circle represent the movement of the food rewards rolling down and the area where the rewards land.

The apparatus (see Fig. 3) was composed of a plastic folding table with a square wooden plank clamped to the top. At one end of the plank a transparent plastic bin was taped so that it could be lifted up or hang down. The bin, at rest, would hang down and remain unmoved on the top of a wooden ramp. A hole big enough to fit blueberries was drilled on the back side of the bin so that when at rest on the ramp, the experimenter could place five blueberries into the bin. A thin purple rope was tied to the far end of the plastic bin and was routed back to the opposite end of the wooden plank. This was set up so that pulling on the purple rope would reliably lift the plastic bin, so blueberries could fall down the wooden ramp and be easily accessible for subjects to obtain. The extreme end of the rope was attached to the mesh of the enclosure. To allow reaching and pulling the rope, we attached a small, handheld, opaque white handle (a PVC pipe). At the right tension, pulling on the handle would reliably lift the plastic bin. The handle could contain a single blueberry inside depending on the condition presented. We used two handles of the same dimensions and appearance to avoid contamination of blueberry leftovers after the trial.

The table with wooden plank would be set up at a distance so that it could not be grabbed by subjects and the ramp was placed underneath so that blueberries would roll down and land in front of the enclosure gate. E2 would then distract the two subjects to an opposite or adjacent side of the subjects’ enclosure with a handful of cereal pieces (in some occasions E2 had to use blueberries to facilitate E1 actions) while E1 tied the end of the purple rope with the handle onto the mesh gate of the enclosure, roughly at the experimenter height, approximately 2 m to the right or left. The distance and location of the rope was kept constant for all trials of each dyad; however, because the enclosures differed in layout, the rope would go to the most convenient side. This way, we ensured that the rope had proper tension to be pulled by gibbons and lift the plastic bin as well as be distant enough from the ramp so that a subject could not easily pull on the rope and obtain food from the ramp at the same time.

### Procedure

Individual solo pre-testing of the mechanism of the apparatus was not possible because the separation of the dyads was prohibited. However, gibbons had had experience with ropes before as part of their enrichment and several individuals had participated in pilot sessions where they had to pull from different ropes and handles.

Three conditions were tested: direct food test condition, indirect food test condition and no food control condition. In the direct food test condition, the following procedure was performed. E1 would place five blueberries in the plastic bin on the apparatus. To gain the attention of the subjects, E1 would call the subjects names and show the food, if they were not already focused on the food/experimenter. Once both subjects had observed the five blueberries placed in the plastic bin, E1 would squeeze a single blueberry on top of the handle, so that the blueberry would be clearly visible. The rope and handle would be set up so that the handle was just far enough from the enclosure (at approximately 30–50 cm) in order for subjects to need to pull on the rope to obtain access to the handle and blueberry. Consequently, pulling the rope would also lift the plastic bin and drop five blueberries down the ramp, accessible to subjects. The experimenter would also call the names of the subjects when placing the single blueberry in the handle. A choice was recorded when one of the subjects pulled the rope. If no subject pulled the rope within 90 s, the trial ended and was recorded as no pull. If an experimenter error was made (e.g. the table was not positioned on the right location or some rewards fell on the ground before the handle was pulled), up to 3 repetitions of the trial would be completed. Environmental conditions such as rain would also end test sessions to be continued the next day.

In the indirect food test condition, there was no single blueberry placed in the handle. To compare conditions, we followed the same procedure as in the direct food test condition. Instead of inserting a blueberry inside the handle, we approached it with the fist close and then we touched it with the fingers. In the no food control condition, no blueberries were used in the trial. In order to control for time and actions, we used the same procedure of calling the subjects and touching both the box and the handle.

### Design

The six dyads participated in 6 sessions of 15 trials each on 6 consecutive days (one session per day). Five trials of each condition were presented within a session in a pseudorandomized order where no condition was done more than twice in a row. In total, 540 trials were completed. There were no dropouts or removed subjects. One set of trials for one dyad had to be continued on the next day due to rain. 8 trials were lost due to errors in video recording. One trial was excluded from model 3 due to a failure of the apparatus.

### Coding and statistical analysis

Two cameras on tripods recorded footage concurrently. One was placed to the side of the experimenter in order to capture a wide view of the trials, specifically to show the positions of the subjects, their choices and if they obtained blueberries. The other was placed close to the ramp to accurately count the quantity of blueberries obtained by each subject. For all trials we coded the act of pulling or not pulling and the ID of the puller (actor subject) and non-puller (passive subject in those cases in which one gibbon pulled). We also coded the number of blueberries each subject ate and whether the actor subject ate the blueberry from the handle. Next, we coded whether a passive subject was present in front of the ramp or within one meter from it at the moment the plastic bin was lifted and at the moment the actor arrived at the release location. Additionally, we coded instances of cofeeding and displacements. Cofeeding was coded when individuals feed within a distance of 1 m of one another^67^. Displacements occurred when an individual left her spot due to the partners’ arrival. Additionally, we calculated the latency to pull from the start of the trial (last frame experimenter touches the handle) until the individual releases (opening of the plastic bin).

All analyses were conducted with R statistics (version 3.4.4). We used Generalized Linear Mixed Models (GLMMs) to investigate gibbons’ choices (models 1, models 3–6). Covariates were z-transformed. Every full model was compared to a null model excluding the test variables. We controlled for session and trial number in all our models. We controlled for the length of the dyad in models 1 to 3 given the larger dataset compared to models 4 to 6. In addition, in model 3 (at the individual level) we included individuals’ age and sex as control predictors. When the comparison between the full and the null model was significant, we further investigated the significance of the test variables and/or their interactions. We used the “drop1” function of the *lme4* package^68^ to test each variable significance including interactions between test predictors. Non-significant interactions were removed and a new reduced model was produced when necessary. A likelihood ratio test with significance set at *p* < 0.05 was used to compare models and to test the significance of the individual fixed effects. We ruled out collinearity by checking Variance Inflation Factors (VIF). All VIF values were close to 1 except for age and length of dyad in model 3. The two variables were slightly collinear (i.e. usually older individuals have been together for longer period of time) (maximum VIF value = 3.26). For every model we assessed its stability by comparing the estimates derived by a model based on all data with those obtained from models with the levels of the random effects excluded one at a time. All models were stable. We also fitted a mixed-effects Cox proportional hazards model (Model 2) to analyze gibbons’ latencies to act. For this purpose, we used the “coxme” function from the *coxme* package^69^. The results of Model 2 are reported as hazard ratios (HR). An HR greater than one indicates an increased likelihood of acting (e.g. releasing the rewards) and an HR smaller than 1 indicated a decreased hazard of acting. In addition, to obtain the p-values for the individual fixed effects we conducted likelihood-ratio tests.

The interobserver reliability was great based on the 19% subset of randomized data sessions that were coded by a second rater. Cohen’s Kappa values were calculated to assess the reliability of rather assignments of gibbons’ decisions and actions. Pearson R^2^ values were calculated to assess the reliability of the latencies to manipulate the handle and release the rewards and the quantity of rewards that each individual ate (participation and IDs of actor and passive partner: Cohen’s Kappa = 1; latencies to manipulate the handles: Pearson R^2^ = 0.99; food consumption and number of rewards obtained: Cohen’s Kappa = 1, Pearson R^2^ = 0.96; passive individual in front of the ramp: Cohen’s Kappa = 1; passive individual in front of the ramp by the moment the subject arrived to the rewards’ location: Cohen’s Kappa = 0.8; occurrence of cofeeding or displacements: Cohen’s Kappa = 0.76).

### Ethics approval

The current research was purely behavioral and non-invasive. The current research has been approved by the IACUC committee of the Gibbon Conservation Center (GCC) and complied with the rules of the IACUC office at University of California, San Diego. The current research was carried out in compliance with the ARRIVE guidelines.

### Informed consent

The person in Fig. 3 is the first author of the study and authorizes the consent to publish the image.

## Supplementary Information

Supplementary Information.

## Data Availability

The data is located in the dryad repository: https://datadryad.org/stash/share/vEjtboMhIKHTShoqSF49f_Po2XGZc2avM8PsDRMoLN8.
