# Supplementary material for: Gibbon strategies in a food competition task
Source: Sci Rep. 2021 Apr 29;11:9312. doi: 10.1038/s41598-021-88804-5 (PMC8085081; doi:10.1038/s41598-021-88804-5)
Supplement: Supplementary file 1 — Supplementary Information. [file 41598_2021_88804_MOESM1_ESM.docx]

**Electronic Supplementary Materials**

**Gibbon strategies in a food competition task**

Alejandro Sánchez-Amaro^1^, Robert Ball^1,2^, Federico Rossano^1^

^1^Department of Cognitive Science, University of California San Diego, United States of America

^2^The Graduate Center, City University of New York, United States of America

**Table S1. General information about the apes that participated in the study.**

| **Name** | **Specie** | **Sex** | **Age at testing** | **Pair ID** | **Length of dyad** | **Relationship** |
| --- | --- | --- | --- | --- | --- | --- |
| Chan Thar | Eastern Hoolock | female | 13 | 1 | 0.5 years | Breeding pair |
| Alan | Eastern Hoolock | male | 7 | 1 | 0.5 years | Breeding pair |
| Hmawe Ni | Eastern Hoolock | female | 15 | 2 | 6.5 years | Contracepted |
| U Maung Maung | Eastern Hoolock | male | 18 | 2 | 6.5 years | Housed with contracepted |
| Betty | Eastern Hoolock | female | 20 | 3 | 6.5 years | Contracepted |
| Khin Maung Win | Eastern Hoolock | male | 12 | 3 | 6.5 years | Housed with contracepted |
| Marlow | Siamang | female | 14 | 4 | 3 years | Contracepted |
| U Myint Swe | Eastern Hoolock | male | 10 | 4 | 3 years | Housed with contracepted |
| Violet | Pileated | female | 10 | 5 | 3 years | Contracepted |
| Truman | Pileated | male | 16 | 5 | 3 years | Housed with contracepted |
| Lucia | Northern White Cheeked | female | 10 | 6 | 1.5 year | Breeding pair |
| Canter | Northern White Cheeked | male | 11 | 6 | 1.5 year | Breeding pair |

**Model details**

**Model 1:** In model 1 we analyzed whether their likelihood to act was influenced by the condition presented. The full model included the test predictor condition and the control predictors session, trial number and length of dyad (in years) as fixed effects. The model also included Dyad ID as random effect and all possible random slopes. The comparison between the full and the null model excluding the test predictor condition was significant (GLMM: *χ*^2^ =121.48, df = 2, *p*<0.001, N = 532). We found a main effect of condition suggesting that gibbons were more likely to participate in direct food test trials compared to indirect food test trials and no food control trials, and that they also participated significantly more in indirect food test trials compared to no food control trials.

| Term | Estimate | Standard Error | Chi-square | Degrees of freedom | p-value | CI (95%) of the model |
| --- | --- | --- | --- | --- | --- | --- |
| Intercept | 0.69 | 0.33 | - | - | - | - |
| Condition (no food) | -0.5 | 0.25 | 121.48 | 2 | 0.048 | -0.99/0.002 |
| Condition (direct food test) | 5.51 | 1.15 | 121.48 | 2 | <0.001 | 4.03/14.81 |
| Session | 0.3 | 0.45 | 0.43 | 1 | 0.51 | -0.62/1.23 |
| Trial | -0.23 | 0.13 | 2.98 | 1 | 0.08 | -0.5/0.002 |
| Length of Dyad | 0.24 | 0.31 | 0.61 | 1 | 0.42 | -0.85/0.46 |

**Model 2:** In model 2 we investigated the latencies of gibbons to act. For this model we established a censor to account for trials in which gibbons did not participate after 90 seconds. The censored data represented 28.2% of the total data (150 of 532 trials). The full model included the test variables condition and the control variables were trial, session and length of dyad as fixed effects. Dyad ID was introduced as a random effect. The comparison between the full and the null model was significant (coxme, *χ*^2^  = 210.18, df = 2, p<0.001, N = 532). We found a significant effect of condition indicating that gibbons acted faster in direct food test trials compared to indirect food test trials and no food control trials.

| Term | Hazard Rate (HR) | p-value | CI (95%) of the model |
| --- | --- | --- | --- |
| Condition (no food) | 0.78 | 0.077 | 0.6/1.04 |
| Condition (direct food test) | 4.98 | <0.001 | 3.74/6.29 |
| Session | 1.09 | 0.003 | 1.01/1.14 |
| Trial | 0.97 | 0.03 | 0.96/1 |
| Length of Dyad | 1.04 | 0.47 | 1/1.1 |


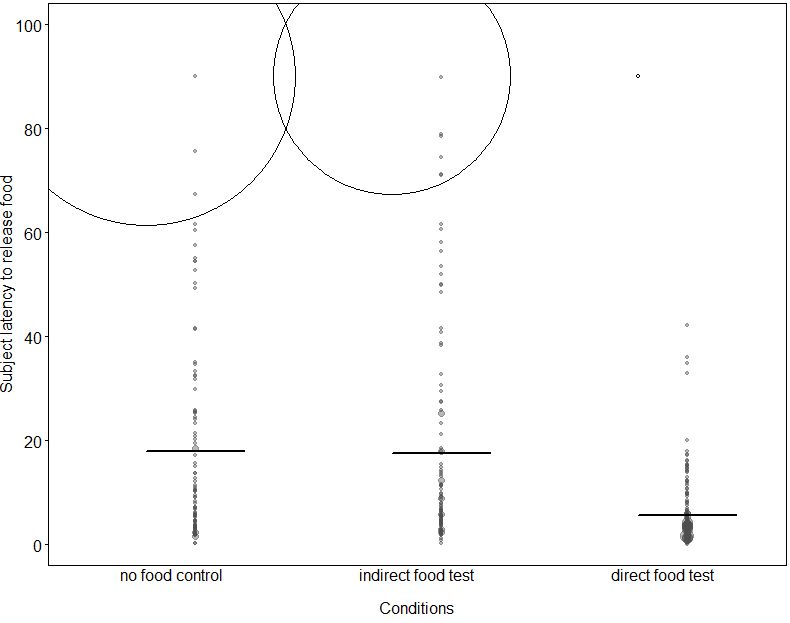


**Figure S1:** Subjects’ latency to release the food across conditions. The dots are density dependent and the white dots represent the amount of times in which subjects did not release the food. That is, they waited over 90 seconds. Horizontal lines represent gibbon mean latencies to pull after discarding trials in which they did not release the food.

**Model 3:** In model 3 we analyzed whether the amount of food obtained was dependent on the type of condition presented and whether individuals volunteered before or not. The full model included the test predictor condition and volunteering act as well as the interaction between the two test predictors, and the control predictors session, trial number, age of participants, sex of participants and length of the dyad as fixed effects. The model also included individual ID as random effect and all possible random slopes. The comparison between the full and the null model excluding the test predictor condition was significant (GLMM: *χ*^2^ =8.89, df = 3, *p* = 0.03, N = 574). We found a significant interaction between the test predictors suggesting that actors—those individuals who had volunteered to release the rewards, obtained more food than passive individuals and the food difference obtained by the actors was higher in indirect food test trials in comparison with direct food test trials.

| Term | Estimate | Standard Error | Chi-square | Degrees of freedom | p-value | CI (95%) of the model |
| --- | --- | --- | --- | --- | --- | --- |
| Intercept | 0.4 | 0.3 | - | - | - | - |
| Condition (direct food test) * Volunteer (direct food test) | -0.89 | 0.35 | 6.89 | 1 | 0.009 | -1.58/-0.24 |
| Session | -0.03 | 0.06 | 0.25 | 1 | 0.61 | -0.14/0.09 |
| Trial | 0.02 | 0.05 | 0.17 | 1 | 0.68 | -0.08/0.12 |
| Age | -0.58 | 0.19 | 7.68 | 1 | 0.006 | -0.98/-0.19 |
| Sex (male) | -0.24 | 0.27 | 0.72 | 1 | 0.39 | -0.8/0.31 |
| Length of dyad | 0.51 | 0.18 | 6.32 | 1 | 0.01 | 0.12/0.94 |

**
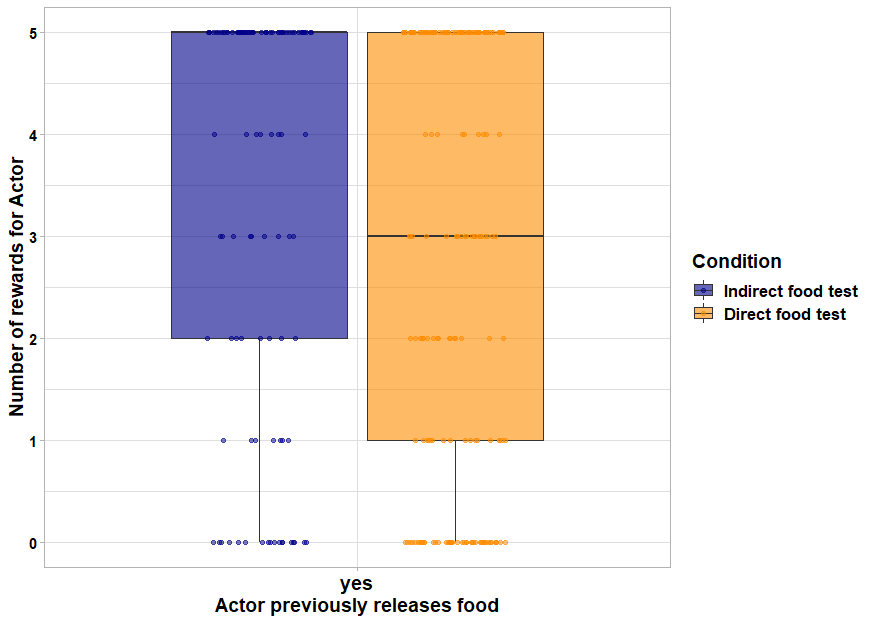
**

**Figure S2:** Boxplot depicting the median and q1 and q3 quartiles of rewards obtained by actors after they had released the food rewards. As you can see, actors usually obtained more than 50% of the released rewards, and they especially maximized their acts in Altruistic trials.

**Model 4:** In model 4 we analyzed whether passive individuals would be in front of the ramp by the time the food was released by the actor. The full model included the test predictor condition and the control predictors session and trial number as fixed effects. The model also included Dyad ID, Actor ID and Passive ID as random effect and all possible random slopes. The comparison between the full and the null model excluding the test predictor condition was not significant (GLMM: *χ*^2^ =0.31, df = 1, *p* = 0.58, N = 287).

**Model 5:** In model 5 we analyzed whether passive individuals would be in front of the ramp by the time the actor arrives to the food location. The full model included the test predictor condition and the control predictors session and trial number as fixed effects. The model also included Dyad ID, Actor ID and Passive ID as random effect and all possible random slopes. The comparison between the full and the null model excluding the test predictor condition was not significant (GLMM: *χ*^2^ =0.69, df = 1, *p* = 0.41, N = 237).

**Model 6:** In model 6 we analyzed whether cofeeding occurrences were influenced by the condition presented passive individuals would be in front of the ramp by the time the food was released by the actor. The full model included the test predictor condition and the control predictors session and trial number as fixed effects. The model also included Dyad ID as random effect and all possible random slopes. The comparison between the full and the null model excluding the test predictor condition was not significant (GLMM: *χ*^2^ =1.58, df = 1, *p* = 0.21, N = 287).
